# Time perspective profile and self-reported health on the EQ-5D

**DOI:** 10.1007/s11136-023-03509-8

**Published:** 2023-09-08

**Authors:** Fanni Rencz, Mathieu F. Janssen

**Affiliations:** 1 Department of Health Policy, Corvinus University of Budapest, 8 Fővám tér, Budapest, 1093 Hungary; 2 Section Medical Psychology and Psychotherapy, Department of Psychiatry, Erasmus MC, Rotterdam, The Netherlands

**Keywords:** Cut-point shift, EQ-5D-5L, Psychological characteristics, Response heterogeneity, Self-reported health, Time perspective

## Abstract

**Objectives:**

Time perspective (TP) is a psychological construct that is associated with several health-related behaviours, including healthy eating, smoking and adherence to medications. In this study, we aimed to examine the associations of TP profile with self-reported health on the EQ-5D-5L and to detect which domains display response heterogeneity (cut-point shift) for TP.

**Methods:**

We conducted a secondary analysis of EQ-5D-5L data from a representative general population sample in Hungary (*n* = 996). The 17-item Zimbardo Time Perspective Inventory was used to measure individuals' TP on five subscales: past-negative, past-positive, present-fatalist, present-hedonist and future. The associations between TP subscales and EQ-5D-5L domain scores, EQ VAS and EQ-5D-5L index values were analysed by using partial proportional odds models and multivariate linear regressions.

**Results:**

Respondents that scored higher on the past-negative and present-fatalist and lower on the present-hedonist and future subscales were more likely to report more health problems in at least one EQ-5D-5L domain (*p* < 0.05). Adjusting for socio-economic and health status, three EQ-5D-5L domains exhibited significant associations with various TP subscales (usual activities: present-fatalist and future, pain/discomfort: past-negative and future, anxiety/depression: past-negative, present-fatalist, present-hedonist and future). The anxiety/depression domain showed evidence of cut-point shift.

**Conclusions:**

This study identified response heterogeneity stemming from psychological characteristics in self-reported health on the EQ-5D-5L. TP seems to play a double role in self-reported health, firstly as affecting underlying health and secondly as a factor influencing one’s response behavior. These findings increase our understanding of the non-health-related factors that affect self-reported health on standardized health status measures.

“*It is far more important to know what person*

*the disease has than what disease the person has*.”

Hippocrates

## Introduction

The belief that psychological dispositions are related to health dates back to Hippocrates (‘the theory of the four humours’) in the 5th century B.C. and has since been generating substantial interest. Over the past decades, an increasing body of evidence demonstrated that personality characteristics are linked to a wide spectrum of health outcomes, including longevity, predicting the development and course of various chronic physical conditions and self-reported health status [1–4]. Time perspective (TP) is a psychological construct that describes how one subjectively focuses on the past, present and future [5]. Some authors consider it to be a trait, while others argue that it is a flexible cognitive structure that may change over the life course, or in response to life events (e.g. traumatic exposure), psychological interventions or social environment [5, 6]. In their seminal work, *Zimbardo and Boyd* distinguished two main aspects of TP, the directionality of one’s thoughts towards time (i.e. past, present or future orientation) and their emotional valence (i.e. positive or negative) [7]. TP has gained increasing attention in the contexts of health and healthcare over the past 30 years. Prior work suggests that persons with past negative view are more likely to experience depression [7], whereas people having a present TP more commonly report using alcohol, drugs, and tobacco [8]. Future TP, in contrast, demonstrated a positive effect on medication adherence and negative effect on partaking in risky sexual behaviour [9, 10].

A few previous studies using the general population or smaller patient samples identified a relationship between TP and self-reported health as measured by a single item health question, the SF-36, SF-12 and WHOQOL-HIV [11–15]. To date, no studies have investigated the association between TP and self-reported health using the EQ-5D*.* The EQ-5D is the most widely used generic preference-accompanied health status measure with a variety of economic (e.g. cost-utility analysis) and non-economic applications (e.g. observational clinical studies, clinical trials, population health surveys and measuring health inequalities) [16–20]. Previous streams of research with the EQ-5D mostly concentrated on the associations between self-reported health and certain personality traits and lifestyle-related attitudes. In these studies, self-reporting less health problems was related to conscientiousness and internal locus of control, while neuroticism, openness, type D personality, ‘live-for-today’ and ‘unconfident fatalist’ attitudes were related to reporting more health problems on the EQ-5D [21–25].

A major measurement issue related to self-reporting own health is that, in addition to the probable link between different psychological factors and health outcomes; for example, as a result of variation in health behaviours or lifestyle choices, psychological characteristics such as TP profile, may also lead to systematic variations in self-reporting own health across respondents with the same health status. It is therefore possible that two people with different psychological traits and the same health status perceive and rate their health differently. This latter variation is commonly referred to as response heterogeneity [26], which may lead to differential item functioning in health status measures [27]. Guided by the framework outlined by *Lindeboom and van Doorslaer*, two forms of response heterogeneity may be distinguished: cut-point shift and index shift [26]. Cut-point shift occurs when the relative positions of the level thresholds change for certain subgroups of respondents directly influencing the shape of the distribution of responses [28]. Index shift refers to a parallel shift in all of the reporting thresholds for certain subgroups of respondents that leads to a shift in the distribution of responses either to the right or left [28]. An extensive body of studies provided evidence of the presence of cut-point or index shift in self-reported health mainly using a single health question [26, 28–30]; however, none of these have investigated individuals’ psychological characteristics as a source of response heterogeneity in self-reported health.

This study seeks to explore the possible link between individuals’ TP profile and self-reported health on the five dimensions of EQ-5D-5L, EQ VAS and index values. We aim to go beyond merely demonstrating the association between TP and self-reported health by attempting to detect which EQ-5D-5L domains possibly display response heterogeneity for TP. Among the two forms of response heterogeneity, our sample enabled to investigate the presence of cut-point shift. We hypothesized that respondents with future, present-hedonistic and past-positive TP reported fewer health problems and respondents with present-fatalistic and past-negative TP reported more health problems [11, 12]. We expected that the pain/discomfort and anxiety/depression domains would be more likely to exhibit response heterogeneity for TP given the more subjective nature of these domains [31].

## Methods

### Study design and population

We conducted a secondary analysis of the cross-sectional data of the EQ-5D-Y-3L (youth) valuation study in Hungary [32]. Respondents were recruited from a large online panel in April and May 2021. The target population for the online panel survey was the Hungarian adult general population aged 18 years or over, and quota sampling methods were used to achieve a representative sample in terms of gender and age (across seven age groupings: 18–24, 25–34, 35–44, 45–54, 55–64, 65–74 and 75+). Ethical approval to conduct the data collection was granted by the Research Ethics Committee of the Corvinus University of Budapest (no. KRH/31/2021). All respondents entering the survey were asked to provide informed consent. To ensure high quality of DCE responses, two quality control criteria were used in the survey regarding completion time and dominant pairs [32]. Respondents that failed to meet either one or both of these quality control criteria were excluded and did not continue with the remaining sections of the survey. Participants that successfully met both quality control criteria proceeded to complete the EQ-5D-5L, 17-item Zimbardo Time Perspective Inventory (ZTPI) and socio-demographic and health-related questions in a fixed order. For the latter, a list of 12 common chronic health conditions was provided for respondents. The question specifically asked respondents to report those health conditions that had been diagnosed by a physician.

### EQ-5D-5L

The EQ-5D-5L is a generic preference-accompanied health status measure that comprises two parts, a descriptive system and a vertical visual analogue scale (EQ VAS) ranging from ‘the worst imaginable health state’ (0) to ‘the best imaginable health state’ (100) [33]. The descriptive system is composed of the following five health domains: mobility, self-care, usual activities, pain/discomfort and anxiety/depression. Each domain has five response levels: no problems (1), slight problems (2), moderate problems (3), severe problems (4) and extreme problems/unable to (5). These five domains describe overall 3125 unique health profiles, with 11111 being the best (full health) and 55555 being the worst possible health state (pits). Index values (i.e. utilities) may be assigned to each profile using a value set that reflects societal preferences. In this study, we computed index values using the Hungarian EQ-5D-5L value set that had been developed using composite time trade-off method [34].

### 17-item Zimbardo Time Perspective Inventory (ZTPI)

**Fig. 1:**
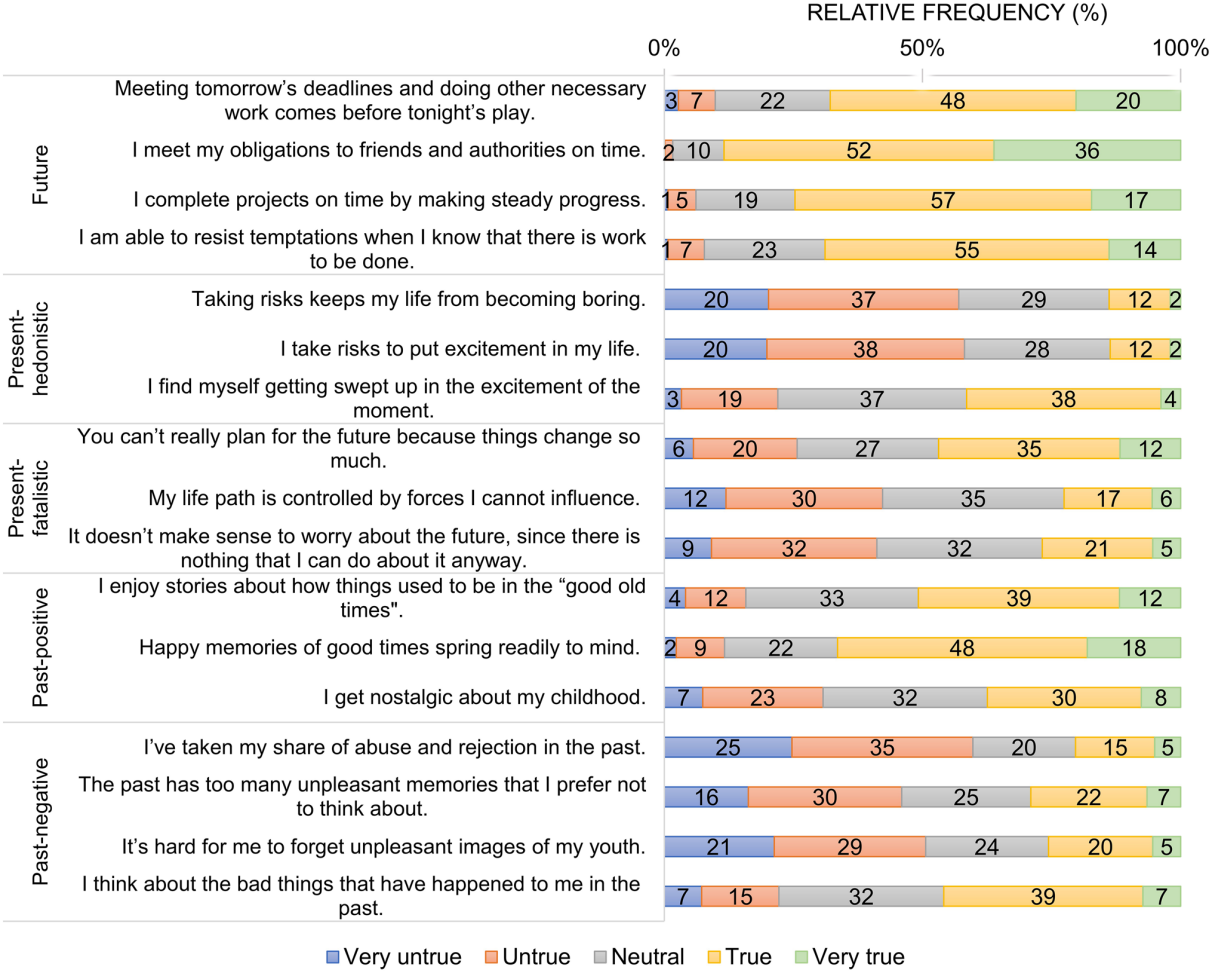
Distribution of responses on the 17-item Zimbardo Time Perspective Scale. Note that the original order of items was reorganised according to subscales for this figure. Figures may not add up to 100% due to rounding

To measure respondents’ TP profile, we used the validated Hungarian version of the 17-item ZTPI that is a shorter version of the original 56-item questionnaire [7, 35]. ZTPI is a multidimensional TP scale that is based on the considerations proposed by *Zimbardo and Boyd* [7]. Figure 1 presents the 17 items of the scale, with each being represented by a statement and assessed on a five-point scale with the endpoints of ‘very untrue’ and ‘very true’. Item scores were summed into subscale scores (past-negative, past-positive, present-fatalistic, present-hedonistic and future) following the official scoring of ZTPI (range of subscale scores 1–5, where a higher score indicates more of the trait being measured).

### Statistical analyses

There were no missing values as all questions were mandatory in the online survey. Descriptive statistics were used to provide an overview of the characteristics of the study population. Mean, standard deviation (SD), median, interquartile range, minimum and maximum were computed for continuous variables (EQ VAS, EQ-5D-5L index values and each TP subscale).

#### Partial proportional odds models: exploring response heterogeneity

We adopted an analytical strategy that aims to test the equivalence in response level thresholds controlling for a variety of individual characteristics, such as socio-demographics and health status [26, 29, 30]. We treated EQ-5D-5L domain scores as ordinal data due to the hierarchy of response levels. The associations between TP subscales and EQ-5D-5L domain scores were analysed using partial proportional odds models [36]. The self-care domain was omitted from the analysis due to limited variability of responses. For the other four domains, responses were collapsed into three categories (no problems, slight problems and moderate-to-extreme problems) to account for the low number of respondents reporting severe or extreme health problems. The three categories were divided by two response thresholds: level 1 vs. levels 2–5 (‘no problems’ vs. ‘slight-to-extreme problems’) and levels 1–2 vs. levels 3–5 (‘no or slight problems’ vs. ‘moderate-to-extreme problems’). The five ZTPI subscale scores, four socio-demographic characteristics (age, gender, education, income) and 12 health condition groups were included in the models as independent variables. These latter were considered as proxies for ‘true’ underlying health status. For all independent variables, the proportional odds assumption was tested using Brant test [37]. The model was sequentially refitted until no variables complied with this assumption. We report the results as odds ratios (ORs) and their 95% confidence intervals. Independent variables that satisfy the proportional odds assumption have a single OR for both response thresholds. Whereas, independent variables not meeting the proportional odds assumption have different ORs for the threshold of ‘no problems’ vs. ‘slight-to-extreme problems’ relative to ‘no or slight problems’ vs. ‘moderate-to-extreme problems’ providing evidence of response heterogeneity (cut-point shift).

#### Multivariate linear regressions

Multivariate linear regressions were performed to investigate the association between TP subscales and EQ VAS and EQ-5D-5L index values. Two separate regressions were run for both outcomes of interest to explore the contribution of TP profile to the explained variance in EQ VAS and EQ-5D-5L index value. In the first models (‘without TP’), EQ VAS and EQ-5D-5L index were regressed on four socio-demographic variables (age, gender, education, income) and 12 chronic health condition groups. In the second models (‘with TP’), the five ZTPI subscale scores were also added to the regression as independent variables in addition to respondents’ socio-demographic characteristics and chronic conditions. To ease interpretation of the coefficients, ZTPI subscale scores were rescaled to range from 0 to 4 before the regression analyses. The presence of heteroscedasticity was confirmed by the Breusch-Pagan test [38]. Robust standard errors were used to correct for any heteroscedasticity. The ‘without TP’ and ‘with TP’ models were compared with regard to the explained variance (*R*^2^ statistic). All analyses were performed in Stata 14 and *p*-values <0.05 were considered statistically significant.

## Results

### Characteristics of the study population

Out of the 1251 participants, 255 (20.4%) did not meet either one or both quality control criteria in the DCE and were consequently excluded from the study. The final sample consisted of 996 respondents and showed an excellent representativeness for gender and age groups. There was a higher proportion of highly educated respondents compared to the adult general population in Hungary (Table 1). The majority reported overall good health status with mean EQ VAS of 78.03 and EQ-5D-5L index of 0.919 (Table 2). Overall, 72, 93, 80, 53 and 67% had no problems with mobility, self-care, usual activities, pain/discomfort and anxiety/depression, and 38% of the sample reported to be in full health (11111).

**Table 1:** Characteristics of the study population

Variables	Reference population^a^	Total sample (*n* = 996)		Variables	Reference population^a^	Total sample (*n* = 996)	
	%	*n*	%		%	*n*	%
Age (years)				Gender			
18–24	10	103	10	Female	53	522	52
25–34	15	157	16	Male	47	474	48
35–44	20	195	20	Education			
45–54	16	167	17	Primary school or less	45	219	22
55–64	17	172	17	Secondary school	33	366	37
65–74	13	134	13	College/university degree	31	411	41
75+	10	68	7	EQ-5D-5L domains			
Household’s per capita net monthly income (HUF)				Mobility			
Quintile 1 (<= 87500.50)	n/a	161	16	No problems	n/a	721	72
Quintile 2 (87500.51–131250.25)	n/a	154	15	Slight problems	n/a	198	20
Quintile 3 (131250.26–175000.33)	n/a	145	15	Moderate problems - unable to	n/a	77	8
Quintile 4 (175000.34–225000.33)	n/a	165	17	Self-care			
Quintile 5 (225000.34+)	n/a	162	16	No problems	n/a	930	93
Don't know/refused to answer	n/a	209	21	Slight problems	n/a	44	4
Chronic health conditions^b,c^				Moderate problems - unable to	n/a	22	2
None	52	461	46	Usual activities			
Allergy	15	160	16	No problems	n/a	800	80
Anxiety	n/a	78	8	Slight problems	n/a	146	15
Asthma	5	56	6	Moderate - extreme problems	n/a	50	5
Cancer	2	33	3	Pain/discomfort			
Cardiovascular disease	>8	120	12	No problems	n/a	526	53
Depression	8	53	5	Slight problems	n/a	380	38
Diabetes	9	103	10	Moderate - extreme problems	n/a	90	9
Gastrointestinal disease	n/a	75	8	Anxiety/depression			
Hypertension	31	305	31	No problems	n/a	664	67
Musculoskeletal disease	>20	239	24	Slight problems	n/a	250	25
Osteoporosis	6	30	3	Moderate - extreme problems	n/a	82	8
Skin disease	n/a	78	8	11111 (full health)	n/a	378	38

^a^Reference values: Hungarian Central Statistical Office: Microcensus 2016

^b^Reference values: Hungarian Central Statistical Office: Health at a glance, 2019

^c^*n*=19 don’t know/refused to answer

Figures may not add up 100% due to rounding. n/a = not available

**Table 2:** Descriptive statistics of EQ VAS, EQ-5D-5L index and ZTPI subscale scores

Measure	Theoretical range	Observed range	Mean	SD	Median	Q1–Q3
EQ VAS	0–100	1–100	78.03	17.22	81	70–90
EQ-5D-5L index	−0.848 to 1	−0.393 to 1	0.919	0.130	0.957	0.907–1
ZTPI future	1–5	1.75–5	3.89	0.55	4.00	3.50–4.25
ZTPI present-fatalistic	1–5	1–5	2.94	0.83	3.00	2.33–3.58
ZTPI present-hedonistic	1–5	1–5	2.65	0.78	2.67	2.00–3.00
ZTPI past-positive	1–5	1–5	3.40	0.81	3.33	3.00–4.00
ZTPI past-negative	1–5	1.5–4.5	2.88	0.50	3.00	2.50–3.25

*EQ VAS* EuroQol visual analogue scale, *ZTPI* 17-item Zimbardo Time Perspective Inventory

The distribution of responses on each ZTPI item is presented in Fig. 1. The item ‘I meet my obligations to friends and authorities on time’ received the highest proportion of affirmative responses (true or very true: 88%), while the disapproval rate (very untrue or untrue) was the highest for the statement ‘I’ve taken my share of abuse and rejection in the past’ (60%). With respect to TP subscales, the highest mean scores were found for the future subscale (3.89), followed by the past-positive (3.40), while the lowest were observed for the present-hedonistic subscale (2.65) (Table 2).

### The association between EQ-5D-5L domain responses and TP

As hypothesized, after adjusting for socio-demographic characteristics and health status, respondents that scored higher on the past-negative and present-fatalistic and lower on the present-hedonistic and future subscales were more likely to report more health problems in at least one EQ-5D-5L domain (Table 3). Three EQ-5D-5L domains exhibited significant associations with various TP subscales (usual activities: present-fatalistic and future [range ORs: 0.60–1.26], pain/discomfort: past-negative and future [range of ORs: 0.69–1.47], anxiety/depression: past-negative, present-fatalistic, present-hedonistic and future [range of ORs: 0.42–2.05]). The mobility domain showed no association with TP profile.

**Table 3:** Partial proportional odds models of the association between time perspective and EQ-5D-5L domains (odds ratio and 95% CI)

	Mobility	Usual activities	Pain/discomfort	Anxiety/depression
Intercept	0.05 (0.01–0.31)**	0.24 (0.03–1.79)	0.44 (0.09–2.01)	0.20 (0.04–1.12)
Time perspective (ZTPI subscales)				
Future				
Level 1 vs. Levels 2–5	0.76 (0.56–1.02)	0.60 (0.43–0.84)**	0.69 (0.54–0.89)**	0.75 (0.57–0.99)*
Levels 1–2 vs. Levels 3–5				0.42 (0.26–0.69)**
Present-hedonistic				
Level 1 vs. Levels 2–5	0.98 (0.79–1.22)	1.00 (0.79–1.28)	0.96 (0.8–1.15)	0.90 (0.73–1.10)
Levels 1–2 vs. Levels 3–5				0.58 (0.40–0.86)**
Present-fatalistic	1.14 (0.93–1.4)	1.26 (1.00–1.58)*	1.14 (0.96–1.35)	1.59 (1.31–1.92)***
Past-positive	1.11 (0.89–1.39)	1.09 (0.85–1.39)	1.05 (0.88–1.25)	0.96 (0.80–1.16)
Past-negative	1.07 (0.76–1.49)	0.89 (0.62–1.29)	1.47 (1.12–1.94)**	2.05 (1.51–2.78)***
Age (years)	1.03 (1.02–1.04)***	1.01 (0.99–1.02)	0.99 (0.98–1.00)	0.97 (0.96–0.98)***
Gender (ref: male)	0.95 (0.68–1.33)	1.58 (1.08–2.29)*	1.56 (1.19–2.05)**	1.27 (0.94–1.71)
Education (ref: primary)				
Secondary	1.00 (0.66–1.52)	0.89 (0.56–1.40)	0.76 (0.53–1.09)	0.92 (0.61–1.39)
Tertiary	1.00 (0.64–1.56)	0.84 (0.52–1.38)	0.79 (0.54–1.16)	1.02 (0.67–1.57)
Income (ref: quintile 1)				
Quintile 2	0.98 (0.58–1.65)	0.94 (0.54–1.63)	1.35 (0.85–2.15)	0.93 (0.56–1.56)
Quintile 3				
Level 1 vs. Levels 2–5	0.83 (0.46–1.50)	0.91 (0.50–1.68)	1.60 (0.99–2.60)	1.19 (0.70–2.01)
Levels 1–2 vs. Levels 3–5	1.58 (0.72–3.47)			
Quintile 4	0.83 (0.47–1.45)	0.44 (0.23–0.85)*	1.01 (0.63–1.64)	0.75 (0.44–1.28)
Quintile 5	0.71 (0.39–1.30)	0.60 (0.31–1.16)	1.31 (0.80–2.16)	1.24 (0.72–2.11)
Don't know/refused to answer	0.72 (0.42–1.25)	0.58 (0.33–1.05)	1.03 (0.66–1.62)	0.81 (0.50–1.33)
Chronic conditions (ref: none)				
Allergy				
Level 1 vs. Levels 2–5	1.47 (0.95–2.28)	2.09 (1.33–3.27)**	0.80 (0.54–1.19)	0.90 (0.60–1.36)
Levels 1–2 vs. Levels 3–5			1.53 (0.86–2.72)	
Anxiety				
Level 1 vs. Levels 2–5	1.75 (0.92–3.32)	1.48 (0.73–2.99)	2.18 (1.23–3.87)**	8.77 (4.92–15.65)***
Levels 1–2 vs. Levels 3–5		0.46 (0.15–1.40)		
Asthma	0.94 (0.49–1.78)	1.83 (0.96–3.46)	1.58 (0.88–2.83)	0.79 (0.4–1.57)
Cancer				
Level 1 vs. Levels 2–5	1.52 (0.72–3.20)	1.35 (0.60–3.03)	1.62 (0.79–3.31)	0.99 (0.41–2.40)
Levels 1–2 vs. Levels 3–5				3.24 (1.01–10.39)*
Cardiovascular disease				
Level 1 vs. Levels 2–5	1.39 (0.85–2.27)	2.26 (1.43–3.58)**	2.24 (1.48–3.38)***	1.30 (0.81–2.10)
Levels 1–2 vs. Levels 3–5	2.92 (1.63–5.24)***			
Depression				
Level 1 vs. Levels 2–5	1.55 (0.73–3.29)	3.25 (1.49–7.09)**	2.7 (1.34–5.44)**	4.83 (2.38–9.80)***
Levels 1–2 vs. Levels 3–5		8.67 (3.26–23.07)***		
Diabetes				
Level 1 vs. Levels 2–5	1.20 (0.74–1.96)	1.59 (0.93–2.72)	1.13 (0.71–1.79)	1.66 (0.97–2.84)
Levels 1–2 vs. Levels 3–5				4.24 (1.95–9.21)***
Gastrointestinal disease	0.61 (0.33–1.13)	0.90 (0.47–1.72)	1.32 (0.79–2.19)	2.52 (1.48–4.29)**
Hypertension	1.72 (1.20–2.48)**	1.64 (1.09–2.47)*	1.61 (1.17–2.23)**	1.17 (0.81–1.71)
Musculoskeletal disease	8.09 (5.69–11.50)***	4.23 (2.88–6.22)***	4.40 (3.17–6.13)***	1.12 (0.77–1.63)
Osteoporosis	1.11 (0.50–2.44)	1.29 (0.55–3.04)	1.64 (0.78–3.45)	0.92 (0.39–2.18)
Skin disease				
Level 1 vs. Levels 2–5	0.78 (0.44–1.39)	0.67 (0.35–1.28)	1.10 (0.67–1.8)	0.58 (0.31–1.08)
Levels 1–2 vs. Levels 3–5				1.89 (0.83–4.34)
Model fit	χ^2^(28) = 348.86, *p *< 0.001, Pseudo *R*^2^ = 0.2326	χ^2^(28) = 242.15, *p *< 0.001, Pseudo *R*^2^ = 0.2000	χ^2^(27) = 273.02, *p *< 0.001, Pseudo *R*^2^ = 0.1486	χ^2^(31) = 329.07, *p* < 0.001, Pseudo *R*^2^ = 0.2008

Note that modelling was not possible for the self-care dimension due to limited variability in responses. Variables that meet the proportional odds assumption exhibit a consistent odds ratio across response thresholds, i.e. comparing level 1 vs. levels 2–5 to levels 1–2 vs. levels 3–5. Conversely, variables that do not satisfy the proportional odds assumption demonstrate different odds ratios between the ‘level 1 vs. levels 2–5’ and the ‘levels 1–2 vs. levels 3–5’ thresholds, indicating the presence of response heterogeneity (cut-point shift).

ZTPI = 17-item Zimbardo Time Perspective Inventory

Level 1 = no problems, level 2 = slight problems, level 3–5 = moderate-to-extreme problems.

^*^ *p* < 0.05; ** *p* < 0.01; *** *p* < 0.001

Several TP subscales, socio-demographic and health status characteristics were found to be in a significant association with one or more EQ-5D-5L domains without evidence of cut-point shifting. For every one-year increase in age, the odds of reporting a one-level higher severity of problems was 1.03 (95% CI 1.02–1.04) for mobility and 0.97 (95% CI 0.96–0.98) for anxiety/depression. Women were 1.58 (95% CI 1.08–2.29) and 1.56 (95% CI 1.19–2.05) times more likely to report a one-level higher severity of problems with usual activities and pain/discomfort than men. Education was not associated with any EQ-5D-5L domain scores, but a higher level of income was related to a lower likelihood of reporting a one-level higher severity of problems with usual activities. The presence of different chronic conditions tended to increase the probability of reporting more problems in each EQ-5D-5L domain. Notably, the highest odds ratios were related to the association between having been diagnosed with anxiety and the anxiety/depression domain (OR 8.77, 95% CI 4.92–15.65) and having musculoskeletal disease and the mobility domain (OR 8.09, 95% CI 5.69–11.50).

### Response heterogeneity

The anxiety/depression domain showed evidence of cut-point shift as demonstrated by the distinct ORs between the ‘no problems vs. slight-to-extreme problems’ and the ‘no or slight problems vs. moderate-to-extreme problems’ thresholds (Table 3). Individuals with higher present-hedonistic or future TP subscale scores were less likely to report moderate-to-extreme problems compared to no or slight problems (present-hedonistic: OR 0.58, 95% CI 0.40–0.86 and future: OR 0.42, 95% CI 0.26–0.69) relative to slight-to-extreme problems compared to no problems (present-hedonistic: OR 0.90, 95% CI 0.73–1.10 and future: OR 0.75, 95% CI 0.57–0.99). Age, gender and education showed no evidence of cut-point shift. One income quintile demonstrated cut-point shift for mobility; however, both separate coefficients were insignificant. An array of chronic condition categories indicated cut-point shift (mobility: cardiovascular diseases, usual activities: anxiety and depression, pain/discomfort: allergy, anxiety/depression: cancer, diabetes, skin disease). Note that only four of these seven chronic condition groups had a statistically significant association with the respective EQ-5D-5L domains.

### The association between TP and EQ VAS and EQ-5D-5L index values

In the first EQ VAS model (‘without TP’), respondents with higher income had slightly higher EQ VAS scores and eight of 12 chronic health conditions were associated with a significant decrease in EQ VAS scores ranging from hypertension (2.55) to depression (10.42) (Table 4). In the second model (‘with TP’), after including respondents’ TP subscale scores in addition to their socio-demographic characteristics and health status, four of the five TP subscales had a significant effect on EQ VAS scores. A one-point increase in the past-negative and present-fatalist subscale scores, all else equal, decreased the EQ VAS score by 2.70 and 2.58 (*p* < 0.05). By contrast, a one-point increase in the future and present-hedonistic subscale scores, all else equal, resulted in a 3.00 and 1.25 increase in EQ VAS score (*p* < 0.05). Respondents’ TP profile (including all five TP subscale scores) increased the explained variance in EQ VAS score from 26.6% (‘without TP’) to 30.2% (‘with TP’).

**Table 4:** OLS regression of the association between time perspective and EQ VAS and EQ-5D-5L index values (regression coefficients and standard errors)

Variables	EQ VAS ‘without TP’	EQ VAS ‘with TP’	EQ-5D-5L index ‘without TP’	EQ-5D-5L index ‘with TP’
Intercept	79.478 (2.43)***	78.979 (4.166)***	0.934 (0.019)***	0.938 (0.031)***
Time perspective (ZTPI subscale score-1)				
Future	–	2.996 (0.935)**	–	0.016 (0.006)**
Present-hedonistic	–	1.246 (0.619)*	–	0.003 (0.005)
Present-fatalistic	–	−2.575 (0.639)***	–	−0.015 (0.005)**
Past-positive	–	0.259 (0.647)	–	−0.001 (0.004)
Past-negative	–	−2.700 (0.98)**	–	−0.009 (0.007)
Age (years)	0.013 (0.033)	0.011 (0.033)	0.000 (0.000)	0.000 (0.000)
Gender (ref: male)	0.426 (0.982)	0.635 (0.968)	−0.006 (0.008)	−0.005 (0.008)
Education (ref: primary)				
Secondary	2.363 (1.454)	2.058 (1.407)	0.008 (0.011)	0.006 (0.011)
Tertiary	0.915 (1.459)	0.216 (1.419)	0.014 (0.01)	0.009 (0.01)
Income (ref: quintile 1)				
Quintile 2	1.343 (2.002)	1.116 (1.954)	0.013 (0.015)	0.011 (0.015)
Quintile 3	1.781 (1.938)	0.989 (1.877)	0.014 (0.015)	0.009 (0.014)
Quintile 4	4.736 (1.864)*	3.966 (1.798)*	0.027 (0.014)	0.023 (0.014)
Quintile 5	4.042 (1.883)*	2.742 (1.832)	0.020 (0.014)	0.012 (0.013)
Don't know/refused to answer	4.353 (1.765)*	3.683 (1.704)*	0.025 (0.013)	0.021 (0.013)
Chronic conditions (ref: none)				
Allergy	0.144 (1.347)	0.111 (1.353)	−0.004 (0.01)	−0.005 (0.01)
Anxiety	−7.949 (2.129)***	−7.140 (2.141)**	−0.081 (0.019)***	−0.078 (0.019)***
Asthma	−4.411 (2.138)*	−3.726 (2.189)	−0.018 (0.02)	−0.015 (0.02)
Cancer	−9.753 (4.185)*	−8.918 (4.123)*	−0.014 (0.025)	−0.009 (0.024)
Cardiovascular disease	−8.388 (1.831)***	−8.673 (1.781)***	−0.070 (0.019)***	−0.071 (0.018)***
Depression	−10.416 (2.643)***	−9.385 (2.642)***	−0.101 (0.028)***	−0.095 (0.027)**
Diabetes	−6.190 (1.74)***	−6.293 (1.686)***	−0.031 (0.017)	−0.032 (0.017)
Gastrointestinal disease	−2.527 (1.932)	−2.184 (1.877)	−0.013 (0.017)	−0.011 (0.017)
Hypertension	−2.548 (1.208)*	−2.600 (1.202)*	−0.026 (0.009)**	−0.026 (0.009)**
Musculoskeletal disease	−7.316 (1.339)***	−7.039 (1.337)***	−0.075 (0.01)***	−0.074 (0.01)***
Osteoporosis	−7.123 (3.686)	−6.844 (3.639)	−0.004 (0.024)	−0.003 (0.023)
Skin disease	−0.567 (1.738)	−0.341 (1.786)	0.004 (0.014)	0.006 (0.014)
Model fit	F(21, 974) = 12.04 (*p *< 0.001), *R*^2^ = 0.266	F(26, 969) = 12.51 (*p *< 0.001), *R*^2^ = 0.302	F(21, 974) = 8.25 (*p *< 0.001), *R*^2^ = 0.309	F(26, 969) = 7.66 (*p *< 0.001), *R*^2^ = 0.326

*EQ VAS* EuroQol visual analogue scale, *TP* time perspective, *ZTPI* 17−item Zimbardo Time Perspective Inventory

^*^ *p *< 0.05; ** *p *< 0.01; *** *p* < 0.001

In the first EQ-5D-5L index model (‘without TP’), no socio-demographic characteristics were associated with index values; however, five of 12 chronic health conditions were resulted in a significant decrease in index values ranging from hypertension (0.026) to depression (0.101) (Table 4). In the second model (‘with TP’), after including respondents’ TP subscale scores in addition to their socio-demographic characteristics and health status, two TP subscales had a significant effect on EQ-5D-5L index values. A one-point increase in the present-fatalistic and future TP subscale scores, was associated with a decrease of 0.015 and an increase of 0.016 in EQ-5D-5L index, all else equal (*p* < 0.05). Respondents’ TP profile increased the explained variance in EQ-5D-5L index from 30.9% (‘without TP’) to 32.6% (‘with TP’).

## Discussion

This study contributes to the growing literature on the link between psychological dispositions and self-reported health on the EQ-5D. Using a large general population sample from Hungary, it provides an insight into the association between individuals’ TP profiles and self-reported health on the EQ-5D. Three EQ-5D domains (usual activities, pain/discomfort and anxiety/depression) as well as the EQ VAS and EQ-5D index values were associated with respondents’ TP profile. Furthermore, we demonstrated the presence of response heterogeneity in the anxiety/depression domain; the probability of reporting more problems in this domain decreased with having more future and present-hedonistic characteristics. As such, this is the first study that identified response heterogeneity on the EQ-5D arising from individual psychological factors. Other authors have used item response theory, Rasch-analysis, Mantel-Haenszel statistics and ordinal logistic regressions, and reported response heterogeneity (or differential item functioning) on the EQ-5D mainly across geographical regions, countries, age groups, sexes, ethnicities, patients vs. proxies and clinically relevant patient groups (e.g. types of cancer or psychosis) [39–46].

Multiple TP subscales were significantly associated with EQ-5D-5L and EQ VAS values. However, the overall impact of TP on EQ VAS and EQ-5D-5L values appears to be relatively small. Findings of a large systematic review suggest that personality characteristics account for varying proportions of health (ranging from 0 to 39%), depending on the health status measure used [4]. In our study, TP explained 3.6% of the variance of EQ VAS and 1.7% of EQ-5D-5L index values. Although these findings are in the range of those reported in the abovementioned review, they fall towards the lower end. It is worth noting that the percentage of explained variance in EQ-5D-5L index values also depends on the value set used. It is likely that using a value set of another country, where anxiety/depression has a larger weight compared to the Hungarian value set, would result in slightly higher explained variance.

Our findings suggest that the impact of certain TP scales, such as future or present-fatalistic, on index values and EQ VAS scores may approximate previously reported MID estimates (0.03–0.10 for the EQ-5D-5L index and 7–11 for EQ VAS) [47–55]. For example, compared to a respondent scoring the minimum on the future TP subscale, a respondent scoring one, two, three or four has, on average, higher EQ-5D-5L index values by 0.016, 0.032, 0.048 and 0.064 and higher EQ VAS scores by 3, 6, 9 and 12 points, respectively.

Respondents’ TP profile and a few chronic condition groups seem to display cut-point shift, a form of response heterogeneity. It is important to stress that for variables not producing any cut-point shift, but being significantly related to self-reported health (e.g. future TP to usual activities and pain/discomfort), an index shift may still occur. In our analytical framework, we accounted for ‘true’ health status by controlling for respondents’ chronic health conditions; however, response heterogeneity may also affect these variables through false reporting [56]. Future research is recommended to use different approaches (e.g. anchoring vignettes, performance measurements, objective clinical variables and item response theory) to isolate index shift as a reporting behaviour from variations in underlying health status [57–62].

Another noteworthy finding from this study is that the EQ-5D showed no evidence of cut-point shift by age, gender and education. Notwithstanding, some domains exhibited significant associations with age or gender that may signal a possible index shift. This is in line with prior work on response heterogeneity on the EQ-5D, whereby older respondents were more likely to report problems with mobility and less likely with anxiety/depression [41, 45]. Even though we cannot rule out the possibility of having more mobility problems with age after controlling for specific chronic health conditions, it may also be possible that these findings are attributable to an index shift. Similarly, our findings suggest a possible index shift on the usual activities and pain/discomfort domains by gender, whereby women were more inclined to report problems than men. In a previous study with cancer patients, the mobility and usual activities domains showed large- and medium-size response heterogeneity by gender [41]. Among the two forms of response heterogeneity distinguished in our analytical framework, index shift is less concerning than cut-point shift due to its linear nature [26].

Possessing more future and present-hedonistic traits may be seen as desirable qualities leading to less health problems, whereas individuals with more past-negative and present-fatalistic characteristics appear to report more health problems. These results are broadly consistent with those of previous studies that identified an association between TP profile and self-reported health measured by various instruments [11–13, 15]. As argued above, these associations must be treated with caution as they are presumably a result of both response heterogeneity and true health effects. A possible explanation for the latter is that TP profile has been found to be related to a number of health behaviours, such as exercising, alcohol, tobacco and substance use, attendance at health screenings and adherence to medications [8, 10, 63–65]. The association between health outcomes and TP profiles is further supported by evidence of the effectiveness of TP-based psychological interventions, such as ‘Time Perspective Therapy’, which have successfully improved mental health in patients with posttraumatic stress disorder [66].

Our findings may have wider implications for patient management, clinical trials and economic evaluations. It seems that non-health factors, such as TP profile may affect one’s ‘true’ health as well as response behaviour on the EQ-5D. Understanding the relationship between TP and health status may help to identify barriers in treatment adherence and to improve patient self-management. Further research is needed to examine whether psychological characteristics, such as TP profile, may be considered a potential source of systemic differences between the treatment and control groups in clinical trials. Lastly, considering that the EQ-5D index values are used to estimate quality-adjusted life years, individual TP may also represent an uncertainty on the results of cost-effectiveness analyses and healthcare decisions based thereon. Exploring the potential impact of respondents’ TP on health preferences in valuation studies is another an important direction for future research.

This study has a number of limitations. First, we used a general population sample, and therefore, there was less variability in respondents’ health status that motivated us in collapsing response levels and excluding self-care from the domain-specific analyses. Secondly, more abundant information about the clinical status of respondents (e.g. severity/stage, symptoms, limitations in functioning) could have been useful to more adequately adjust our models for ‘true’ health. Thirdly, selection bias may have occurred not only because of the online mode of administration that excluded people without internet access or sufficient computer literacy, but also due to the study design. During the DCE tasks, 255 respondents were excluded based on quality control criteria, such as providing inconsistent responses on the dominant pairs [32]. As these tasks may be viewed as some kind of logical or cognitive test, it is likely that respondents with somewhat higher cognitive abilities accomplished them and therefore were selected to the final sample. Fourthly, the original 56-item ZTPI questionnaire has been subject to some criticisms with regard to its construct validity and dimensionality [5, 67]. In our study, we used a 17-item short version of this scale that performed well in most psychometric tests in an earlier study in Hungary [35]. However, its face validity may still be questioned; for example, some of the items may rather capture beliefs, values or preferences that do not directly relate to TP and therefore may represent alternative psychological constructs [5, 68, 69].

In conclusion, this is the first study to explore the association between individuals’ TP and self-reported health on the EQ-5D and also the first to identify response heterogeneity (cut-point shift) stemming from psychological characteristics on the EQ-5D. It seems that psychological factors may play a double role in self-reported health, firstly as affecting underlying health and secondly as a factor influencing one’s response behavior. These findings increase our understanding of the non-health-related factors that affect self-reported health on standardized health status measures.

## Data Availability

All data of this study are available from the corresponding author upon reasonable request.
